# Sporadic lymphangioleiomyomatosis in a man with somatic mosaicism of *TSC2* mutations, a case report

**DOI:** 10.1093/qjmed/hcad235

**Published:** 2023-10-16

**Authors:** Y Wang, D Hu, Y Liu, L Yang, J Huang, J Zhou, L Guo, X Fan, X Huang, M Peng, C Cheng, W Zhang, R Feng, X Tian, S Yu, K -F Xu

**Affiliations:** Department of Pulmonary and Critical Care Medicine, State Key Laboratory of Complex Severe and Rare Diseases, Peking Union Medical College Hospital, Chinese Academy of Medical Sciences and Peking Union Medical College, Beijing, China; Department of Pulmonary and Critical Care Medicine, State Key Laboratory of Complex Severe and Rare Diseases, Peking Union Medical College Hospital, Chinese Academy of Medical Sciences and Peking Union Medical College, Beijing, China; McKusick-Zhang Center for Genetic Medicine, State Key Laboratory of Medical Molecular Biology, Institute of Basic Medical Sciences, Chinese Academy of Medical Sciences and Peking Union Medical College, Beijing 100005, China; Department of Pulmonary and Critical Care Medicine, State Key Laboratory of Complex Severe and Rare Diseases, Peking Union Medical College Hospital, Chinese Academy of Medical Sciences and Peking Union Medical College, Beijing, China; Department of Pulmonary and Critical Care Medicine, State Key Laboratory of Complex Severe and Rare Diseases, Peking Union Medical College Hospital, Chinese Academy of Medical Sciences and Peking Union Medical College, Beijing, China; Department of Radiology, Peking Union Medical College Hospital, Chinese Academy of Medical Sciences and Peking Union Medical College, Beijing, China; Department of Dermatology, State Key Laboratory of Complex Severe and Rare Diseases, Peking Union Medical College Hospital, Chinese Academy of Medical Sciences and Peking Union Medical College, National Clinical Center, Beijing, China; Clinical Genome Center, Guangzhou KingMed Diagnostics Group Co., Ltd., Guangdong, China; Clinical Genome Center, Guangzhou KingMed Diagnostics Group Co., Ltd., Guangdong, China; Department of Pulmonary and Critical Care Medicine, State Key Laboratory of Complex Severe and Rare Diseases, Peking Union Medical College Hospital, Chinese Academy of Medical Sciences and Peking Union Medical College, Beijing, China; Department of Pulmonary and Critical Care Medicine, State Key Laboratory of Complex Severe and Rare Diseases, Peking Union Medical College Hospital, Chinese Academy of Medical Sciences and Peking Union Medical College, Beijing, China; Department of Radiology, Peking Union Medical College Hospital, Chinese Academy of Medical Sciences and Peking Union Medical College, Beijing, China; Department of Pathology, State Key Laboratory of Complex Severe and Rare Diseases, Peking Union Medical College Hospital, Chinese Academy of Medical Sciences and Peking Union Medical College, Beijing, China; Department of Pulmonary and Critical Care Medicine, State Key Laboratory of Complex Severe and Rare Diseases, Peking Union Medical College Hospital, Chinese Academy of Medical Sciences and Peking Union Medical College, Beijing, China; Clinical Genome Center, Guangzhou KingMed Diagnostics Group Co., Ltd., Guangdong, China; Department of Pulmonary and Critical Care Medicine, State Key Laboratory of Complex Severe and Rare Diseases, Peking Union Medical College Hospital, Chinese Academy of Medical Sciences and Peking Union Medical College, Beijing, China

Learning points for clinicians Lymphangioleiomyomatosis (LAM) is a rare, multi-system neoplasm that almost exclusively occurs in women with presentation of sporadic or tuberous sclerosis complex (TSC)-associated type. Sporadic LAM in male patients as well as its mechanisms has been rarely fully described. We reported a 32-year-old Chinese male with histopathologically and genetically confirmed sporadic LAM. A novel *TSC2* mutation (c.4494_4545del) was found in different specimen with a various mutation frequency. Somatic mosaic *TSC2* mutations (c.4494_4545del) resulted in a multi-system involvement including pulmonary LAM, kidney and liver angiomyolipomas and sclerotic bone lesions. Meanwhile, a somatic 111 bp exonic duplication involving exon 4 of *TSC2* and a somatic *TSC1* mutation (c.1525C>T) were also detected in renal angiomyolipomas.

Thus, gene sequencing in various tissue should be considered to establish the diagnosis in those who lack other presentations of TSC.

Lymphangioleiomyomatosis (LAM) is a rare, multi-system neoplasm that almost exclusively occurs in women.^1^ LAM can be divided into sporadic and tuberous sclerosis complex (TSC)-associated types. LAM in male patients has been rarely described.^2^ Here, we report a man with histopathologically diagnosed sporadic LAM caused by somatic mosaicism for novel *TSC2* mutations.

A 32-year-old Chinese man was evaluated for a 10-year history of intermittent chest pain and mild dyspnea during the past 11 months. The patient received a nephrectomy 10 years ago to remove an angiomyolipoma (AML) in his right kidney. He reported no prior history of pulmonary diseases, neurologic disorders or skin lesions. He was a former cigarette smoker until 1 year ago. There was no family history of pulmonary bullae or TSC, and his two children were in normal health condition. On physical examination, the patient presented with normal male appearance and no dermatological evidence of TSC.

**Figure 1.**
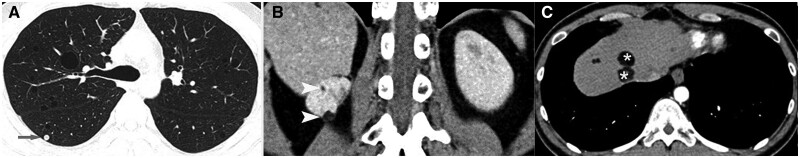
Radiological manifestation of the chest, kidney and liver. (**A**) High-resolution computed tomography at 2.00 mm slice thickness shows uniformly distributed thin-walled cysts throughout the lung parenchyma bilaterally, with scattered small solid nodules (arrow). (**B**) Post-operative coronal contrast-enhanced CT shows angiomyolipomas in kidney (arrowhead). (**C**) Contrast-enhanced CT section shows multifocal angiomyolipomas in liver (asterisk).

On evaluation, his chest high resolution computed tomography (CT) showed characteristic pattern of numerous, diffuse, thin-walled cysts in both lungs, which was suggestive of LAM and several small nodules (Figure 1A). Abdominal CT and magnetic resonance imaging (MRI) showed multiple AMLs in the liver and the right kidney (Figure 1B and C). No abnormalities were observed in the brain MRI. Skeleton on CT demonstrated six sclerotic bone lesions in pelvis. Pulmonary function test showed forced expiratory volume at 1 s (FEV_1_) 85% predicted, forced vital capacity 97% predicted, total lung capacity 85% predicted and diffusion capacity for carbon monoxide 76% predicted. Peripheral blood chromosome analysis displayed a 46, XY karyotype. Systemic evaluation on eyes, mouths and heart did not find abnormalities. Serous vascular endothelial growth factor D was within normal range (467 pg/ml).

A video-assisted thoracoscopic lung biopsy revealed smooth muscle-like cell clusters around the cystic lesion with positive immunohistological staining of HMB45, estrogen receptor and melan-A on pathology. The kidney specimen obtained from previous surgery was re-evaluated, demonstrating AML with positive staining of HMB45 and α smooth muscle actin.

The *TSC1* and *TSC2* exonic profile of the patient’s blood sample, lung tissue and renal AML specimen was explored using next generation sequencing and illustrated in Table 1. A novel *TSC2* mutation (c.4494_4545del), interpreted as a pathogenic one based on American College of Medical Genetics (ACMG) guidelines, was found in different specimen with a various mutation frequency. The variant was detected in the AML with a frequency of 21.51%, much higher than that in the pulmonary lesions (0.29% and 2.12%). Meanwhile, a somatic 111 bp exonic duplication involving exon 4 of *TSC2* and a somatic *TSC1* mutation (c.1525C>T) were also detected in renal AMLs. The 3 mutations mentioned above were undetectable in the peripheral blood mononuclear cell on a coverage of 100 000× in both *TSC1* and *TSC2*.

**Table 1.** Allele frequency of the *TSC1* and *TSC2* mutation in kidney and lung

Sample	Gene	Mutation	Allele frequency (%)
Renal AMLs	*TSC2*	c.4494_4545del	21.51
Renal AMLs	*TSC1*	c.1525C>T	2.29
Renal AMLs	*TSC2*	Exon 4 CNV	
Lung parenchyma	*TSC2*	c.4494_4545del	0.29
Lung cysts	*TSC2*	c.4494_4545del	2.12
Blood	Negative		

AML: angiomyolipoma; CNV: copy number variation; TSC: tuberous sclerosis complex.

The mutation detected in both lung and kidney tissue was not detected from the patient’s peripheral blood mononuclear cell sample with high depth sequencing, indicating a somatic variant and therefore supporting the diagnosis of sporadic LAM instead of TSC. Mosaicism of the *TSC2* mutation explained both the multi-system involvement and the discrepant variant frequencies in different specimen. In a previously reported male LAM patient, an extreme mosaicism of *TSC2* 2320delA was found.^3^ In our case, a novel frameshift mutation (c.4494_4545del) in *TSC2* was found in the renal AML with a mutant allele frequency of 22.15% and in lung at a lower frequency of 0.29–2.12%. The 111 bp exonic duplication of exon 4 in *TSC2* might be considered as a second hit although its pathogenicity however needs further investigation. The pathogenicity of the somatic *TSC1* mutation (c.1525C>T) is also needed to be investigated.
